# Clinical Outcomes of Gonioscopy-Assisted Transluminal Trabeculotomy in Eyes After Failed Trabeculectomy

**DOI:** 10.3390/jcm14186524

**Published:** 2025-09-17

**Authors:** Agnieszka Ćwiklińska-Haszcz, Dominika Wróbel-Dudzińska, Ewa Kosior-Jarecka

**Affiliations:** Department of Diagnostics and Microsurgery of Glaucoma, Medical University of Lublin, 20-079 Lublin, Poland; agnieszka@cwiklinscy.pl (A.Ć.-H.); dominikawrobeldudzinska@umlub.pl (D.W.-D.)

**Keywords:** GATT, MIGS, glaucoma surgery, failed trabeculectomy

## Abstract

**Background**: This study aims to present the clinical outcomes, safety profile, and mid-term results of gonioscopy-assisted transluminal trabeculotomy (GATT) in eyes with failed trabeculectomy. **Methods**: The studied group consisted of 62 patients after failed trabeculectomy. All patients underwent GATT, as a standalone procedure or in combination with cataract surgery (Phaco-GATT), between 2021 and 2023 and had at least 12 months of follow-up. The patients were examined at 1 day, 7 days, 1 month, 3 months, 6 months, and 12 months after surgery, with evaluation of visual acuity, intraocular pressure (IOP), number of antiglaucoma drops, and possible surgical complications. **Results**: The mean IOP before surgery was 37.22 ± 9.07 mmHg, and after the GATT procedure, it significantly decreased to 15.91 ± 5.28 mmHg at the 12-month follow-up. A comparison of patients with one or more previous antiglaucoma procedures showed no differences. A comparison of the degrees of successful canulating and deroofing of Schlemm’s canal revealed no statistical significance concerning IOP values or the number of medications. When comparing the IOP between the patients after standalone GATT and Phaco-GATT, a tendency for higher IOP values in the latter group was observed. In 21 (33.3%) patients, hyphema affecting visual acuity was observed early after surgery, which resolved spontaneously. Six patients (9.7%) needed additional surgery to obtain the target pressure. **Conclusions**: GATT is an effective and safe IOP-lowering surgical option in open-angle glaucoma patients with failed trabeculectomy.

## 1. Introduction

Glaucoma is a group of conditions leading to irreversible sight loss and characterised by progressive loss of retinal ganglion cells [1]. It is one of the most common diseases causing blindness worldwide. In 2020, glaucoma affected over 76 million people, and by 2040, it is expected to be diagnosed in 111.8 million [2]. Although not always elevated, intraocular pressure (IOP) is the only modifiable risk factor in glaucoma treatment that can influence the disease course, as demonstrated by large clinical trials [1]. Moreover, many studies have shown that the successful treatment of glaucoma is based on lowering IOP [3,4] to the target IOP level, which sufficiently decreases the glaucoma progression rate to an acceptable level. The target pressure can be obtained using medical, laser, or surgical treatment.

Trabeculectomy, a golden standard in glaucoma surgery, is an incisional antiglaucoma procedure introduced by Cairns in 1968 [5]. During the surgery, an additional path is created for aqueous humour to leave the eye’s anterior chamber, omitting the natural outflow pathways. Since its introduction, trabeculectomy has been modified with releasable sutures and the additive use of antimetabolites to make the procedure more efficient and safer, with a longer-lasting IOP-lowering effect [6]. Trabeculectomy can obtain IOP in low teen values, which is usually enough to slow glaucoma progression [7]. However, its high efficacy is contrasted by the risk of complications, some of which, usually related to hypotony, are potentially sight-threatening [8]. The final success of trabeculectomy depends not only on the perfect surgical technique but also on the ability to effectively control postoperative healing processes [9]. The late failure of trabeculectomy usually results from scarring within the area of newly created outflow [10]. In cases of primary trabeculectomy failure, revision procedures are usually performed to restore bleb outflow [11]. However, its efficacy is lower compared to primary procedures because each subsequent procedure affecting the conjunctiva and sclera causes excessive scarring, resulting in unrestrained healing [12].

Although trabeculectomy is very effective in decreasing IOP, its safety profile is not always satisfactory. Thus, new surgical procedures have been introduced, focusing on minimising side effects. The techniques in antiglaucoma surgery that are grouped as minimally invasive glaucoma surgery (MIGS) include procedures that spare the conjunctiva and sclera; some of them improve the natural aqueous humour outflow pathways via Schlemm’s canal (SC). Gonioscopy-assisted transluminal trabeculotomy (GATT), proposed by Groover et al. [13], is a bleb-independent ab interno intervention, where the inner Schlemm’s canal wall is removed and the conventional pathway of aqueous humour is promoted by decreasing outflow resistance. Previously published studies have proved that GATT is a primary antiglaucoma procedure effective in lowering IOP in different types of open-angle glaucoma, juvenile open-angle glaucoma, and childhood glaucoma [13,14,15,16,17].

MIGS techniques are usually offered to glaucoma patients as a first-line surgical treatment in cases with mild to moderate stages of the disease. The aim of this study is to evaluate the efficacy and safety of GATT in patients with prior failed primary trabeculectomy.

## 2. Materials and Methods

### 2.1. Studied Group

The studied group consisted of 62 patients after trabeculectomy at the Department of Diagnostics and Microsurgery of Glaucoma, Medical University of Lublin, Poland. All patients who met the inclusion criteria were prospectively included. This study was designed with the approval of the Local Ethics Committee and the Resolution of the Bioethics Committee and performed in accordance with the standards set by the Declaration of Helsinki.

The inclusion criteria were as follows:

- Previously performed failed trabeculectomy;
- Target IOP not obtained after the previous surgical procedures and maximal tolerated antiglaucoma drops;
- Open-angle glaucoma;
- Age over 18 years;
- Written consent agreement to the surgery and to inclusion in this study.

Patients with normal-tension glaucoma were not included in the study group.

At the qualification, the patients underwent detailed anterior and posterior eye segment evaluation with measurement of best-corrected visual acuity, IOP, gonioscopy, and 24-2 perimetry (if BCVA was better than 0.1).

All the patients underwent GATT as a standalone procedure or in combination with cataract phacoemulsification by two experienced surgeons between 2021 and 2023 and had at least 12 months of follow-up. The combined procedure with cataract surgery was performed in 18 eyes (29%), and GATT alone was performed in 44 eyes (71%). Forty patients were diagnosed with primary open-angle glaucoma (POAG) and twenty-two with pseudoexfoliative glaucoma (PEXG). The detailed demographic characteristics of the studied group are shown in Table 1.

The patients were examined 1 day (1 D), 7 days (7 D), 1 month (1 M), 3 months (3 M), 6 months (6 M), and 12 months (12 M) after the surgery. At each visit, slit-lamp examination, IOP (Goldmann applanation tonometry), BCVA (Snellen decimal scale), and binocular ophthalmoscopy were performed. After the surgery, all antiglaucoma medications were withdrawn, and the patients received topical ofloxacin and dexamethasone 5 times a day up to the 10 D visit, when the treatment was gradually reduced up to 1 M, when all postoperative medications were stopped. In cases of hyphema, the patients additionally received 1% tropicamide for 10 days. Surgical success was recorded according to the previously established criteria, and the possible complications were noted. If the success criteria were not met, antiglaucoma drops were introduced, starting with a single medication. The early spike of IOP was considered when IOP extended the success criteria 1 D after surgery; the increase in IOP at 10 D was classified as surgical failure.

Two criteria were established to assess the success of the surgery:

S1 A 30% decrease in IOP as compared to the value measured at the last visit before surgery;
S2 IOP ≤ 18 mm Hg.

Both criteria were reported as a total success (“S1” or “S2”) when they were met without antiglaucoma drops. Qualified success (“QS1” or “QS2”) was noted when the criteria were fulfilled with additional medical treatment. In cases where additional surgical treatment was needed to reach a target IOP level, the procedure was considered a failure.

### 2.2. Surgical Procedure

At the temporal side of the eye, a 1.6 mm clear corneal incision was performed. Cohesive viscoelastic was injected to fill the anterior chamber. Around 1 to 2 clock hours later, goniotomy was performed at the opposite site of the angle. One end of a Prolene 6/0 suture was blunted with cautery and gently inserted into the goniotomy. Along 360 degrees, the Schlemm’s canal was then cannulated. If cannulation in one direction did not fully encircle the Schlemm’s canal’s circumference, from the same goniotomy, cannulation in the other direction was attempted. By pulling both ends of the suture, the inner wall of the Schlemm’s canal was peeled off, causing canal unroofing. The extent of Schlemm’s canal deroofing was noted; the surgery aimed to achieve the maximal possible opening. This was followed by anterior chamber irrigation to wash out the viscoelastic, and intracameral cefuroxime was injected.

In 18 eyes, GATT surgery was combined with phacoemulsification. A standard 2.2 mm clear corneal incision cataract surgery was performed at the temporal side of the eye before GATT. After implanting the posterior chamber intraocular lens, a solution of miochol was injected to constrict the pupil. All steps of GATT were subsequently performed as previously described. Cataract surgeries were performed by AĆH, and GATT procedures were performed by EKJ.

Statistical evaluation of the data was performed using Statistica 13.1 (Polish version, Statsoft Poland, Kraków, Poland). The results were reported mainly as mean ± SD or percentage values. A *p*-value lower than 0.05 was considered statistically significant.

## 3. Results

In the studied group, the mean IOP before surgery was 37.22 ± 9.07 mmHg, and after the GATT procedure, it significantly decreased to 15.91 ± 5.28 mmHg at the 12-month follow-up. When comparing all the postoperative values to the preoperative values, statistical significance was found at all points. Table 2 shows all the postoperative IOP measurements. Moreover, IOP was stable after the procedure at the time of the study (IOP 1 D vs. 12 M; *p* = 0.6095). According to the established criteria of success in 12 M, the success rate was 88.8% for S1 and 90.3% for S2; however, approximately half of the patients needed antiglaucoma medication to fulfil the criteria. At 12 M, observation failures were more frequent in the early postoperative period, with stabilisation after 1 M. The detailed success rate results are presented in Table 3 and Table 4.

A statistically insignificant decrease in BCVA was noted on day 1 after surgery. It was caused by hyphema, which spontaneously resolved in all cases at the 10 D visit. The details of the BCVA changes are presented in Table 5. Preoperatively, the mean MD was −16.63 ± 10.27 dB and did not change during the follow-up period (at 12 M MD −14.38 ± 9.64 dB; *p* = 0.3571). The mean number of antiglaucoma medications taken daily decreased significantly after the surgery at 12 M (2.48 ± 1.06 and 1.45 ± 1.21, respectively; *p* = 0.0002). However, an increase in medications between 1 D and 12 M was noted (Table 6).

In 32 patients, only trabeculectomy was performed before the GATT procedure. Thirty patients had more than one procedure (including trabeculectomy with one or two bleb revisions). Analysis of IOP decrease between these two groups revealed no statistical significance after 12 months (12 M 15.08 ± 5.82 vs. 16.85 ± 4.78 *p* = 0.4519), and the percentage decrease values were 56.6% and 58.0%, respectively. There was also no statistical significance in the number of medications taken daily after 12 months (1.58 ± 1.31 in group one vs. 1.33 ± 1.15 in group two; *p* = 0.6250).

According to the extent of the opening of Schlemm’s canal, patients were divided into two groups: one with an opening of 180 degrees or less and one with an opening of more than 190 degrees. Comparison of the degrees of successful Schlemm’s canal cannulation and deroofing revealed no statistical significance concerning the IOP values or the number of medications at any follow-up point. The IOP comparison results are shown in Table 7. Additionally, the number of previous antiglaucoma surgeries did not influence the IOP results (Table 8).

Comparing the IOP values between the patients after standalone GATT and Phaco-GATT revealed a tendency for higher IOP values in the latter group at 10 D and 6 M. The detailed results of the comparison are shown in Table 9.

In 21 (33.3%) patients, hyphema affecting visual acuity was observed at 1 D, which spontaneously resolved at the 10 D visit without additional treatment. Six patients (9.7%) required additional surgery to reach the target pressure: two patients required an Ahmed valve; two patients required cyclophotocoagulation of the ciliary body; one patient required needling revision; and one patient required trabeculectomy.

## 4. Discussion

Late failure of trabeculectomy with increased IOP is caused by excessive scarring. In our department, in cases of unsuccessful trabeculectomy, needling revision is typically performed. The technique is carried out with a bent 5G needle to disrupt formed synechiae at the level of conjunctiva, Tenon’s capsule, and intrascleral; additionally, the needle is introduced into the anterior chamber via a fistula [12]. However, such intervention subsequently activates the healing processes, finally leading to the renewal of inflammatory scarring at the level of the conjunctiva and Tenon’s capsule. A longer period between primary trabeculectomy and revision can lead to better IOP-lowering results for the latter, probably due to the superior attenuation of inflammation. However, clinically successful IOP lowering is not always obtained with a revision procedure, and it is best to plan the next surgery based on the different mechanisms of IOP-lowering action.

The main site of outflow resistance for aqueous humour from the eye’s anterior chamber in conventional outflow pathways is thought to be located in the region of the inner wall of SC and the juxtacanalicular tissue (JCT) [18]. The high IOP in POAG is caused by an increase in the outflow resistance at this level. However, on the other hand, elevated IOP during the course of glaucoma also causes multiple changes within SC [19,20]: studies evaluating POAG patients showed decreased SC cross-sectional area, perimeter, and length [21,22,23]. Histopathologic studies revealed increased collapse and narrowing of collector channels (CCs) and intrascleral veins, adhesion of SC endothelium to CC orifice walls, and herniation of JCT with blockage of CC orifices [24]. Additionally, a decrease in SC size after trabeculectomy was described and attributed to a decline in meshwork perfusion and extracellular material accumulation, leading to increased outflow resistance [25]. Considering the mentioned SC changes during the course of glaucoma, SC-based surgeries are believed to lose efficacy in advanced stages of glaucoma. Moreover, it was long thought that SC-based procedures are not effective in chronic adult glaucoma, especially in cases of previous surgery omitting natural outflow pathways, such as trabeculectomy. However, some studies show GATT IOP-lowering efficacy in advanced glaucoma [16,26,27], and, on the other hand, the expansion of SC after trabeculectomy is reported [28].

GATT is based on removing the inner wall of Schlemm’s canal and, as a result, decreasing outflow resistance. Its efficacy depends on the proper function of distal outflow pathways, which, unfortunately, cannot be plausibly evaluated before the surgery. Only a few studies have evaluated the efficacy of GATT in patients after incisional, bleb-forming surgeries. Grover et al. [29] described 35 patients who underwent GATT after primary incisional surgery and found that the procedure was safe and successful in treating 60% to 70% of open-angle patients with prior incisional glaucoma surgery. Cubuk et al. [30] reported a case series study that included 26 eyes of patients who underwent GATT using the 5/0 Prolene suture to treat medically uncontrolled moderate to advanced glaucoma despite a previous trabeculectomy surgery. In this group, the average baseline IOP was 25.3 ± 5.4 (16–45) mmHg, which significantly declined after the procedure to IOP values that were lower in PEXG than in POAG patients (13.4 mmHg vs. 17.1 mmHg). To obtain such an IOP, most patients needed antiglaucoma medication, but the GATT procedure enabled a significant reduction in their numbers. Wang et al. [31] described 44 eyes of 35 patients (21 with juvenile-onset open-angle glaucoma and 14 with adult-onset POAG). Of the patients, 79.5% underwent one previous incisional surgery and 20.5% underwent two prior surgeries. In the case series, IOP decreased from 27.4 ± 8.8 mm Hg on 3.6 ± 0.7 medications preoperatively to 15.3 ± 2.7 mm Hg on 0.5 ± 0.9 medications at the 24-month visit. The complete and qualified success rates were 60.9% and 84.1%, respectively. Siddhartha et al. [32] studied 27 eyes after a failed trabeculectomy and 3 eyes after a failed glaucoma drainage device, which underwent GATT (21 eyes) or Phaco-GATT (9 eyes). In this group post-GATT, the IOP decreased from 27.1 ± 7 mmHg to 16.9 ± 6 mmHg at the end of 15 months. At postoperative 1 year, the probability of complete success was 20% for an IOP criterion of both 21 and 16 m Hg, but the qualified success probability at 1 year was 82% for an IOP of 21 mmHg and 57% for an IOP of 16 mmHg.

The mean IOP value in our patients at the 12 M visit was 15.91 mmHg, which was significantly lower than the preoperative values. The rates of success for precluded criteria were about 90%; however, almost half of the patients needed medical treatment to obtain them. Only six patients needed additional surgery. In the case of GATT, as a minimally invasive procedure, the IOP-lowering results are encouraging. Additionally, the results of our study seem to be more favourable than the previous reports. We believe that the higher rate of success in our group is related to two possible reasons. The first is the high baseline IOP, which makes it easier to obtain a 30% decrease in IOP, which is usually difficult with SC surgery in cases with IOP lower than 20 mmHg. The second possible reason may be the midterm follow-up. On the other hand, most surgical failures after GATT are observed within 3 months after the surgery and are probably related to permanent distal outflow impairment.

In our group of patients, high preoperative IOP with a mean value of more than 37 mmHg on maximal tolerated medical therapy drew attention. It was originally believed that in patients with such high IOP values, SC and natural outflow pathways are not to be preserved, and the results of MIGS are unsatisfactory. Our previous study [33], similar to this one, shows that despite high preoperative IOP, the 12 M IOP is about 15 mmHg, not worse than the results typically obtained with this technique. Additionally, we divided the patients into two groups: one group with one failed trabeculectomy and a second group with patients after trabeculectomy with additional IOP-lowering interventions. Although the preoperative IOP was significantly higher in the latter group, there was no difference in the postoperative IOP values. These results show that the GATT technique may be effectively applied in quite a broad spectrum of open-angle glaucoma patients, and high IOP values are not a bad prognostic factor.

Our study is the first to show that in GATT after trabeculectomy, the limited removal of the SC inner wall is also sufficient to successfully decrease IOP. The localisation of collector channels is not obvious and predictable in patients before the surgery. Studies of the pig eye showed that the main localisation of the outflow is the nasal-inferior quadrant. However, in humans, the localisation of the outflow is more variable and differs not only between subjects but also between the two eyes of one patient. Additionally, in the human eye, low-flow regions could become high-flow, and vice versa, based on need [34]. This suggests that as the opening of Schlemm’s canal is increased, a lower IOP may be obtained. This was not confirmed in previous studies evaluating GATT as primary surgery, which surprisingly showed that results of GATT and hemiGATT were similar [35,36,37,38]. Moreover, hemiGATT performed in superior or inferior hemispheres did not differ in postoperative IOP [39]. Our results also confirm this for refractory glaucoma: the 180-degree deroofing of Schlemm’s canal allowed us to obtain a 12 M IOP similar to total Schlemm’s canal deroofing. The other argument for hemiGATT is that it causes less hyphema and accelerates eye recovery compared to 360 GATT. The exception is congenital glaucoma, where the maximal Schlemm’s canal opening is related to a lower IOP [40].

Previous trabeculectomy poses the risk that advancing the catheter in SC might be difficult or even impossible in the sclerostomy area; the advancement of the catheter may also be prematurely terminated with its appearance in the anterior chamber. However, GATT surgery is usually possible by changing the direction of the suture or making other adjustments to the technique. Depending on the position of the sclerotomy against SC, we usually choose the direction of the first catheter implementation to be opposite to the localisation of the sclerotomy to obtain the maximal part of the circumference catheterised at the first attempt.

In our group of patients, in cases of lens opacification, GATT surgery was combined with cataract surgery. In the case of many MIGS surgeries, adding cataract removal enables lower postoperative IOP. However, few studies compare GATT and Phaco-GATT. Zhang et al. [41] compared postoperative IOP reduction between a GATT alone group and a GATT combined with cataract extraction group. The number of postoperative glaucoma medications was higher in the GATT alone group, and the reoperation rate was also higher in the GATT group. In a retrospective analysis of 64 eyes, Yozgat et al. [42] also confirmed that Phaco-GATT and standalone GATT demonstrated comparable efficacy and safety over a two-year period. On the other hand, Bozkurt et al. [43] showed that GATT alone has a superior lowering effect on IOP than combined surgery, but there was no difference in the final IOP values. A possible explanation for the worse results of Phaco-GATT is the more inflammatory reaction caused by the combined procedure. A similar tendency for the inferior IOP-lowering effect of the combined procedure was also observed in our study, but a further evaluation of a larger patient group is required.

Moreover, cataract surgery has a different influence on glaucoma patients depending on the history of trabeculectomy. Teckan et al. [44] analysed the effect of uneventful cataract surgery on IOP in PEX glaucoma with and without previous trabeculectomy with adjacent MMC. In the trabeculectomised eyes, an increase in IOP and the mean number of medications was significant. In our group of patients, mitomycin was typically applied on sponges during primary trabeculectomy and injected during the revision procedure. In this study, during GATTs combined with cataract surgeries, no injections of antimetabolite or anti-VEGF were applied to protect the bleb. The further decrease in bleb function caused by cataract surgery may be the reason that the IOP-lowering results were slightly worse in the Phaco-GATT group. The potential influence of adjunctive therapies during Phaco-GATT requires further evaluation.

GATT surgery is a safe procedure. One of its very common complications is hyphema, which is usually self-limiting and transient. In this case series, hyphema spontaneously resolving within 10 D after surgery without IOP spikes was observed in 33% of the patients. Macrohyphema was described as a negative prognostic factor for GATT success [45]. Our studies show that hyphema after GATT is rarely combined with high IOP, and even if it requires surgical removal, IOP remains stable and low. In our group of patients, we did not notice early IOP spikes after the surgery. One possible reason is the maximal removal of viscoelastics before the end of the surgery. This decreases the incidence of IOP spikes but increases the risk of prominent hyphema. However, in our group of advanced glaucoma patients, IOP safety was crucial, and a possible decrease in BCVA was discussed with the patients before surgery. The possible complications reported by other authors, such as Descemet detachment, irydodialisis, ciliochoroidal detachment, corneal oedema, vitreous haemorrhage, malignant glaucoma [46,47], or cystoid macular oedema, were not observed in our studied group.

This study has some limitations. The evaluation of surgical success was performed according to established criteria across all patients. However, in clinical practice, individual values of target IOP are set to monitor each patient. GATT typically allows for lowering IOP to a mid-teen value, which was also shown in this study. This value is not always enough to stabilise glaucoma progression, especially in advanced cases. In our study, we attempted to open SC to the maximal extent; however, this was not always possible. For statistical reasons, the studied group was divided into two groups according to tear extent: more and less than 180 degrees. It is possible that the lower extent of the procedure, especially below 120 degrees, may cause a lower IOP decrease. However, the number of patients with an SC opening extent below one-third of its circumference was too small to conclude. Additionally, in our group, the time between trabeculectomy and GATT was not reported because our study group was heterogeneous regarding the number of procedures. Some patients had undergone additional revision procedures, which may influence the inflammatory status of the anterior chamber and, finally, the GATT results.

To conclude, our study shows that GATT is an effective and safe IOP-lowering procedure applied in open-angle glaucoma patients after unsuccessful trabeculectomy.

## Figures and Tables

**Table 1:** Demographic features of the studied group.

Number of Patients	62
Age	72 ± 12.46 years
Gender	29 F/33 M
Type of glaucoma	POAG 40 PEXG 22
IOP	37.22 ± 9.07 mmHg
MD	−16.63 ± 10.27 dB
Number of medications	2.48 ± 1.06
Mean number of previous surgeries	1.59 ± 0.67
Time from the last incisional surgery	27.82 ± 21.32 months

**Table 2:** Mean IOP at each follow-up.

Preoperative	37.22 ± 9.07 mmHg
1 D	15.27 ± 5.45 mmHg *
10 D	18.83 ± 9.00 mmHg *
1 M	19.24 ± 6.27 mmHg *
3 M	18.05 ± 12.54 mmHg *
6 M	15.82 ± 4.88 mmHg *
12 M	15.91 ± 5.28 mmHg *

* At all postoperative visits, IOP was significantly lower compared to the preoperative values, *p* < 0.0001.

**Table 3:** Success criteria: 30% IOP decrease.

30% IOP Decrease	1 D	10 D	1 M	3 M	6 M	12 M
S	79.0%	56.4%	46.7%	51.6%	54.8%	54.8%
QS	79.0%	61.3%	77.3%	88.8%	91.9%	88.8%
F	21.0%	38.7%	22.7%	11.2%	8.1%	11.2%

**Table 4:** Success criteria: IOP lower than 18 mmHg.

IOP Lower than 18 mmHg	1 D	10 D	1 M	3 M	6 M	12 M
S	88.7%	75.8%	50.0%	51.6%	51.6%	50%
QS	88.7%	80.6%	77.4%	90.3%	93.5%	90.3%
F	11.2%	19.4%	22.6%	9.7%	6.5%	9.7%

**Table 5:** Best-corrected visual acuity during the study.

Best-Corrected Visual Acuity		*p*-Value Compared to Preoperative
Before the surgery	0.48 ± 0.37	x
1 D	0.32 ± 0.31	*p* = 0.008
10 D	0.40 ± 0.3	*p* = 0.0420
1 M	0.46 ± 0.31	*p* = 0.3482
3 M	0.54 ± 0.34	*p* = 0.6896
6 M	0.53 ± 0.33	*p* = 0.8072
12 M	0.47 ± 0.37	*p* = 0.5563

**Table 6:** Number of antiglaucoma medications taken by the patients during the study.

Number of Antiglaucoma Medications	
Before the surgery	2.48 ± 1.06
1 D	0.25 ± 0.74 *
10 D	0.44 ± 0.97 *
1 M	0.84 ± 1.16 *
3 M	1.11 ± 1.18 *
6 M	1.25 ± 1.25 *
12 M	1.45 ± 1.21 *

* At all postoperative visits, the number of antiglaucoma drops was significantly lower compared to the preoperative values, *p* < 0.0001.

**Table 7:** Comparison of IOP values depending on the extent of Schlemm’s canal deroofing.

	90–180 Degrees	190–360 Degrees	*p*-Value
Number of patients	24	38	
Before surgery	37.31 ± 9.02.	37.94 ± 10.17	*p* = 0.8146
Day 1	15.87 ± 5.95	13.47 ± 3.55	*p* = 0.1235
Day 10	18.19 ± 6.93	20.82 ± 13.73	*p* = 0.3190
1 M	19,76 ± 6.75	18.56 ± 5.34	*p* = 0.5232
3 M	18.68 ± 14.43	16.64 ± 5.58	*p* = 0.6105
6 M	16.06 ± 5.27	14.66 ± 3.64	*p* = 0.4630
12 M	15.31 ± 5.16	18.2 ± 5.67	*p* = 0.2871

**Table 8:** Comparison of mean IOP depending on the number of previous antiglaucoma surgeries.

	One Glaucoma Surgery Before GATT	More than One Glaucoma Surgery Before GATT	*p*-Value
Number of patients	32	30	
Before surgery	34.78 ± 7.97	40.12 ± 9.56	*p* = 0.0144
Day 1	14.40 ± 4.18	16.32 ± 6.05	*p* = 0.1504
Day 10	18.91 ± 5.66	18.73 ± 12.03	*p* = 0.9339
1 M	18.59 ± 6.5	20.03 ± 5.99	*p* = 0.3535
3 M	16.60 ± 5.39	19.68 ± 17.4	*p* = 0.3783
6 M	15.94 ± 4.51	15.62 ± 5.52	*p* = 0.8381
12 M	15.08 ± 5.82	16.85 ± 4.78	*p* = 0.4519

**Table 9:** Comparison of IOP values between the GATT and Phaco-GATT groups.

	GATT	Phaco-GATT	*p*-Value
number of patients	44	18	
IOP preoperatively	37.0 ± 9.28	37.88 ± 8.63	*p* = 0.7313
Day 1	15.41 ± 5.76	14.88 ± 4.52	*p* = 0.7318
Day 10	17.66 ± 6.02	22.29 ± 14.36	*p* = 0.0664
1 M	19.46 ± 5.95	18.58 ± 7.25	*p* = 0.6213
3 M	18.76 ± 14.5	16.26 ± 4.83	*p* = 0.5190
6 M	15.07 ± 3.57	17.5 ± 6.92	*p* = 0.1551
12 M	16.52 ± 5.81	14.42 ± 3.64	*p* = 0.3877

## Data Availability

The data are available on request from the corresponding author.
